# Family socioeconomic status and young children digital addiction: a moderated mediation model

**DOI:** 10.3389/fpsyg.2024.1435575

**Published:** 2024-09-27

**Authors:** Huanhuan Li, Mengzhen Luo, Bingyu Duan, Abidan Kawulia, Menglu Su, Huijuan Di

**Affiliations:** ^1^College of Educational Science, Xinjiang Normal University, Urumqi, China; ^2^Department of Preschool Education, Hebei Normal University, Shijiazhuang, China

**Keywords:** family socioeconomic status, parenting styles, digital addiction, young children digital addiction, gender

## Abstract

**Background:**

Presently, the problem of digital addiction in young children is becoming more and more prominent, and digital addiction can cause significant harm to the healthy physical and mental development of young children. A growing body of research suggests that family socioeconomic status and parenting styles are associated with digital addiction. However, little is known about the mediating and moderating mechanisms behind this relationship, and few studies have explored whether this relationship holds in young children populations. Therefore, the present study aimed to investigate whether parenting styles mediate the relationship between family socioeconomic status and young children’s digital addiction and whether young children’s gender moderates this mediation process.

**Methods:**

A cross-sectional study design was used. 403 parents of young children were asked to complete online questionnaires, including the Internet Addiction Test-10 (IAT-10) the Chinese version of the Parenting Style Questionnaire (C-EMBU). The mediation model with moderation was tested using the PROCESS plug-in for SPSS.

**Results:**

(1) Family socioeconomic status is significantly and negatively associated with digital addiction in young children. (2) Parenting styles (emotional warmth and understanding, punishment and harshness) mediate the relationship between family socioeconomic status and young children’s digital addiction. (3) Young children’s gender moderates the relationship between family socioeconomic status and punishment and severity parenting styles, emotional warmth and understanding parenting styles and young children’s digital addiction.

**Conclusion:**

The results indicate that family socioeconomic status can prevent digital addiction in young children through the path of improving parenting styles. However, there is still an overall negative effect of family socioeconomic status on young children’s digital addiction.

## 1 Introduction

With the rapid advancement of China’s informatization work, the “Internet + education” model is developing rapidly, and the Internet penetration rate of underage Internet users has also been further enhanced. By June 2020, the Internet penetration rate for minors exceeded 94 percent, the number of children accessing the Internet reached 183 million, and more than 33 percent of primary schoolchildren used the Internet for the first time before school age (China Communist Youth League, 2021). Over 70% of young children are exposed to and use online media. Among them, 34.53% were 3-year-olds, and 21.13% were 4-year-olds (Tang, 2023). The trend of “under-aging” of Internet use cannot be ignored. Although many studies have pointed to the positive effects of Internet use on the development of cognitive functioning and well-being (Heo et al., 2015; Chopik, 2016; Zhou et al., 2022), inappropriate or excessive use of the Internet can lead to digital addiction, which may result in neurological complications, psychological disorders, and social problems (Cash et al., 2012). Digital addiction is a severe psychological disorder that manifests itself as an individual’s inability to effectively control their use of the Internet and digital media, resulting in an impaired psychological state or functioning (Young, 1996; Young and Rogers, 1998). It has been shown that digital addiction hurts the healthy development of young children, such as reduced visual acuity, delayed physical development, weakened social interaction, slowed cognitive and language development, and reduced family intimacy (Liu et al., 2020). At present, several scholars have conducted in-depth studies on the influencing factors and mechanisms of digital addiction among Chinese adolescents, college students, and older people (Yang et al., 2022; Zhang et al., 2022; Gao et al., 2023; Xie et al., 2023; Wang et al., 2024; Yang et al., 2024). However, no scholars have focused on young children in this area. Therefore, this study takes young children as the research object and explores the mechanisms behind the influencing factors of digital addiction to provide a theoretical basis and practical support for the prevention of young children’s digital addiction and the maintenance of young children’s physical and mental health.

At present, many scholars have explored the influencing factors of digital addiction, among which the factor of family socioeconomic status has attracted much attention. Family socioeconomic status refers to a hierarchical ranking based on the degree of social capital, such as rights and status, available to or controlled by the family, reflecting differences in an individual’s access to actual or potential resources, and the social capital involved generally includes factors such as family members’ educational attainment, income level, and occupational reputation. (Zhang et al., 2007) One study showed a significant positive correlation between family socioeconomic status and digital addiction, as evidenced by the correlation between higher family socioeconomic status and a higher propensity to become addicted to the Internet (Mann, 2006). Another study also showed that students from high family socioeconomic status scored significantly higher on the tendency to digital addiction than students from low family socioeconomic status (Zou et al., 2014). However, some studies pointed to the opposite conclusion that family socioeconomic status has a significant negative correlation with digital addiction (Urbanova et al., 2019). This result may be because an individual is in a high socioeconomic status household, which will reduce their reliance on online social activities. In contrast, households with low socioeconomic status can increase their dependencies on online social activities (Jin et al., 2017). It can be seen that the socioeconomic status of the family is a prominent influence on digital addiction. However, regarding the relationship between family socioeconomic status and digital addiction, Scholars have not yet reached an agreement.

In addition to family socioeconomic status, the relationship between parenting styles and digital addiction has also attracted the attention of scholars. Parenting style is a relatively stable conceptual, behavioral, and emotional expression of parents’ treatment of their children, reflecting the nature of the interaction between parents and children. Its dimensional structure includes emotional warmth and understanding, punishment and harshness, over-interference, favoring subject, denial, and over-protection (Perris et al., 1980). It has been shown that parenting behaviors directly affect the behavioral development of young children (Kanan et al., 2018). Parents who tend to adopt negative parenting styles are more likely to cause their children to develop more psychological and behavioral problems, increasing their risk of digital addiction (Fu et al., 2004). A previous study found significant differences in parenting styles between individuals with and without digital addiction tendencies, such as over-interference, punishment and harshness, and denial (Li and Zhang, 2004). Based on this, another study conducted an in-depth exploration of the relationship between parental parenting styles and digital addiction and found a significant positive correlation between denial and over-interference parenting styles and digital addiction (Yu, 2015). In addition to the parenting styles mentioned above, recent research suggests that over-protection parenting styles are also a significant positive predictor of digital addiction (Guo et al., 2023).

It is worth noting that there is also a significant correlation between family socioeconomic status and parental parenting styles. Research has shown that parents raising their children stems from their expectations of their children, and this behavior varies with changes in the family’s socioeconomic status (Hoff et al., 2002). It is reflected in the fact that parents of lower socioeconomic family status are more likely to use harsh, more authoritarian parenting styles, such as corporal punishment and lack of communication, and parents in families of higher socioeconomic status tend to adopt a caring and understanding parenting style and have more interaction and communication with their children (Shonkoff, 2000). Additionally, it has been pointed out that compared to high-income families, low-income families usually associate media time with family time. Parents of low-income families may feel stressed or overworked, resulting in a poorer response to their children’s needs (Clark, 2013; Livingstone et al., 2015).

Many scholars have focused on the moderating role of gender in the relationship between family socioeconomic status and parenting style, and parenting style and digital addiction. Regarding the relationship between family socioeconomic status and parenting styles, one study found that boys in high family socioeconomic status families were likely to experience less parental rejection and more emotional warmth. At the same time, this association was not significant among girls (Cheng and Wu, 2021). A subsequent study noted that when inequality in family socioeconomic status increases, parents have higher expectations of boys compared to girls and are more inclined to adopt intensive parenting styles, such as authoritative and authoritarian parenting, to increase boys’ expected future earnings in the labor market (Li and Zhu, 2022). In addition, another study shows that the lower the household’s socioeconomic status, the greater the gender inequality in access to resources (Wu, 2012). Specifically, in families of lower socioeconomic status, parents are inclined to satisfy boys’ interests at the expense of girls and tend to use overprotective and spoiled parenting styles for boys. In contrast, girls are more likely to be criticized, harsh, and punished (Zhang and Ma, 2019). Regarding the relationship between parenting styles and digital addiction, previous studies have pointed out that when boys do not receive emotional support from their parents, they develop a psychological sense of “abandonment,” a feeling that is stronger in boys than in girls (Wang, 2008). In order to give vent to their bad moods and relieve their depressed moods, boys choose online games more often to vent and release their repressed moods and anxieties through online games. A subsequent study indicated that parenting styles of monitoring significantly and negatively predicted digital addiction in both boys and girls, constraints significantly and positively predicted digital addiction in boys, and neglect and material rewards significantly predicted digital addiction in girls (Li and Zhou, 2009). In addition, recent research has shown that in adolescent populations, positive parenting styles are significantly more negatively predictive of digital addiction among male parents than among female students (Niu et al., 2023). Other scholars exploring the relationship between negative parenting styles and digital addiction among college students found that gender moderated the relationship between parental rejection and digital addiction, whereas gender did not significantly moderate the relationship between parental over-protection and digital addiction (Guo et al., 2023).

**Figure 1:**
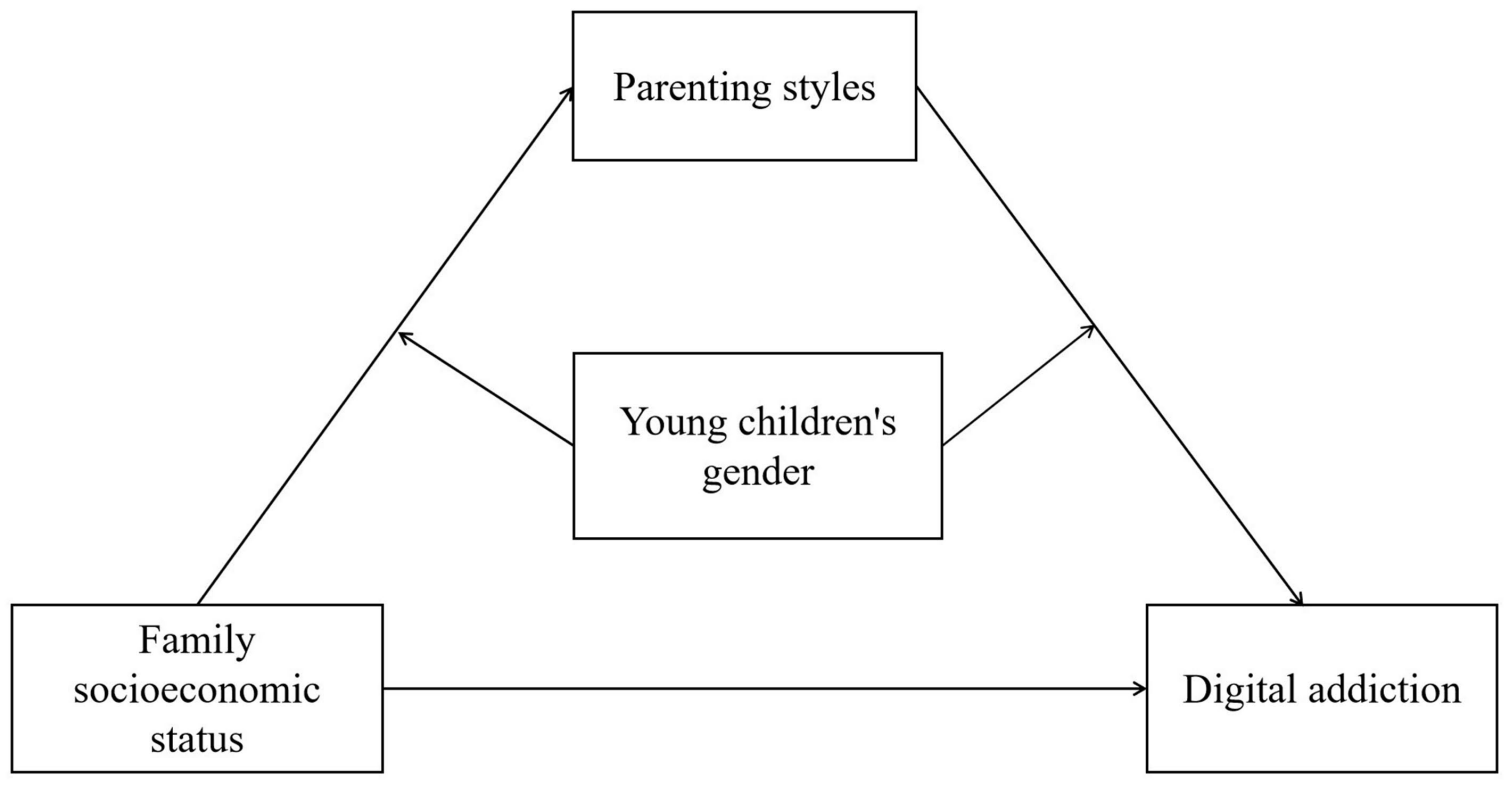
Research hypothesis model.

To summarize, first, previous studies have found that family socioeconomic status and parenting style are essential factors in digital addiction, which provides many references for this study. However, the specific relationship between family socioeconomic status and digital addiction and parenting style and digital addiction is still controversial. Second, many scholars have focused on the moderating role of gender in the relationship between family socioeconomic status and parenting styles, and parenting styles and digital addiction but have not reached uniform conclusions. Third, numerous studies in recent years have pointed out that parenting styles, parent–child relationships, and children’s digital addictions are a dynamic process that changes over time and that there is significant heterogeneity among different groups (Cao and Liu, 2023; Liu et al., 2023; Xie et al., 2023). But most of the existing studies have been conducted on young children, college students, and the elderly population, and there is a lack of attention to digital addiction in the early childhood population. Therefore, in exploring the relationship between family socioeconomic status and young children’s digital addiction, the present study used parenting style as an essential mediating variable and considered the moderating role of gender in the mediating process. Based on that, this study constructs a moderated mediation model to explore the relationship between family socioeconomic status and young children’s digital addiction (see Figure 1). The following four hypotheses were formulated in this study:

*H1:* Young children’s family socioeconomic status is a significant negative predictor of their digital addiction.

*H2:* Parenting style mediates the relationship between family socioeconomic status and young children’s digital addiction.

*H3:* Young children’s gender moderates the relationship between family socioeconomic status and parenting style.

*H4:* Young children’s gender moderates the relationship between parenting style and digital addiction.

## 2 Materials and methods

### 2.1 Participants

The sample of this study using a random sampling approach comprised 415 parents of young children recruited from Urumqi, Xinjiang. After eliminating samples with incomplete information, regular responses, apparently garbled responses, and primary caregivers who were not parents, the actual valid sample size entering the analysis was 403. There were 304 females (75.43%) and 99 males (24.57%), 7 were of them 16–25 years old (1.74%), 206 were of them 26–34 years old (51.12%), 167 were of them 35–45 years old (41.44%), 22 were of them 46–55 years old (5.46%) and one was of them 56–65 years old (0.25%). The gender of the parents’ children was 208 (51.61%) boys and 195 (48.39%) girls, and the age of the children was 3–4 years 147 (36.48%), 4–5 years 94 (23.33%), 5–6 years 133 (33%), 6–7 years 26 (6.45%) and others 3 (0.74%). 140 parents reported their work as general managers, general professional and technical staff, and clerical staff (34.74%). 110 parents were casual workers, unemployed, unemployable, unskilled, and agricultural workers (27.3%). 89 parents’ occupations were manual labor workers (commercial service workers), self-employed workers, skilled workers, and workers of the same grade (22.08%), and 45 parents were engaged in middle managers, middle-level professional and technical staff, and assistant professionals (11.17%). The remaining 19 parents had the occupation of career senior managers (managers), senior professional and technical staff, and professional supervisors (party leaders)(4.71%). The literacy levels of parents represented in the study sample include the following: 1 (0.25%) below the elementary school, 5 (1.24%) in elementary school, 67 (16.63%) in middle school, 90 (22.33%) in high school or junior college, 231 (57.32%) in college (college or bachelor’s degree), and 9 (2.23%) in graduate school and above.

### 2.2 Measures

#### 2.2.1 Digital addiction scale

This study referred to the *Internet Addiction Test* (IAT-10) developed by Young (1996) at the University of Pittsburgh, United States, based on the digital addiction Screening Scale. It mainly deals with the length of time the child uses electronic products, emotions, outcomes, and behavioral manifestations. The questionnaire consists of 10 “yes” or “no” questions and young children who answered “yes” to five or more questions were diagnosed with digital addiction. The Cronbach’s alpha coefficient for this scale in this study was 0.842.

#### 2.2.2 Parenting style scale

Parenting styles were measured using the *Chinese version of the Parenting Styles Questionnaire Questionnaire* (C-EMBU). The questionnaire was developed by Perris et al. (1980) scale and revised by our scholars Yue et al. (1993). Following China’s national conditions, the C-EMBU contained 66 items, of which 58 items used to evaluate the father’s parenting style, consisting of six factors: emotional warmth and understanding, punishment and harshness, over-interference, favoring subject, denial, and over-protection; and 56 items used to evaluate the mother’s parenting style, consisting of five factors: emotional warmth and understanding, over-interference, denial, punishment and severity, and favoring subject. The reliability of the dimensional scales was 0.7 or higher.

### 2.3 Statistical analysis

First, Harman’s one-factor approach has been used to detect systematic errors due to the homogeneity of the environment and the psychology of evaluating the overall expectations of the sample. Second, means, standard deviations and correlations of variables of the present study were reported through SPSS 26.0. Finally, the mediating role of parenting style was tested using the macro program PROCESS v4.1.1. The moderating role of young children’s gender in the first half of the model path and the second half of the model path was analytically tested.

## 3 Results

### 3.1 Common method bias test

Harman’s one-factor approach has been used to detect systematic errors due to the homogeneity of the environment and the psychology of evaluating the overall expectations of the sample. The results showed that of the 14 characteristic root factors extracted, the first common factor accounted for 22.233% of all explanatory variables, less than the 40% determination criterion proposed by Podsakoff et al. Therefore, there is no serious problem of common method bias in the data of this study.

### 3.2 Descriptive statistics and correlation analysis

The means, standard deviations, and correlation coefficients of the variables are shown in Table 1. As can be seen, family socioeconomic status was significantly and negatively associated with digital addiction in young children (*p* < 0.01), emotional warmth and understanding parenting style was significantly and positively related to digital addiction in young children (*p* < 0.01), rejection and denial was negatively associated with digital addiction in young children (*p* < 0.001), and punishment and harshness parenting style was significantly and negatively related to digital addiction in young children (*p* < 0.05), family socioeconomic status was significantly positively correlated with emotional warmth and understanding parenting style with digital addiction in young children (*p* < 0.001), family socioeconomic status was significantly negatively correlated with punishment and harshness parenting style (*p* < 0.01).

**Table 1:** Results of descriptive statistics and correlation analysis for each variable.

Item	*M*	SD	1	2	3	4	5	6	7
Young children’s digital addiction	1.627	0.284	–						
Emotional warmth and understanding	2.942	0.465	0.143**	–					
Over-intervention and over-protection	2.176	0.503	−0.058	0.327***	–				
Rejection and denial	1.508	0.517	−0.181***	0.135**	0.669***	–			
Punishment and harshness	1.756	0.463	−0.121*	0.176***	0.643***	0.838***	–		
Favoring subject	2.623	0.449	0.056	0.592***	0.451***	0.336***	0.386***	–	
Family socioeconomic status	9.75	2.836	−0.160**	0.155**	−0.059	−0.057	−0.157**	0.049	–

*N =* 403; *p < 0.05; **p < 0.01; ***p < 0.001.

### 3.3 Testing for the mediation model

The Bootstrap method provided bias-corrected confidence estimates of the mediating role of parenting styles and young children’s digital addiction and the statistics analyzed by the SPSS macro program PROCESS (Preacher and Hayes, 2004). As shown in Table 2. Family socioeconomic status significantly negatively predicted young children’s digital addiction (*β* = −0.160, *p* < 0.01). After the introduction of mediator variables, family socioeconomic status negatively predicted young children’s digital addiction through emotional warmth and understanding parenting style (*β* = −0.019, *p* < 0.001), and family socioeconomic status negatively predicted children’s digital addiction through punishment and harshness parenting style (*β* = −0.184, *p* < 0.001). Meanwhile, emotional warmth and understanding parenting styles significantly positively predicted young children’s digital addiction (*β* = 0.173, *p* < 0.001), and punishment and harshness parenting styles significantly negatively predicted young children’s digital addiction (*β* = −0.150, *p* < 0.01).

**Table 2:** Results of inter-mediation analysis.

Regression equation		Fitness index			Significance of regression coefficients			
Implicit variable	Independent variable	*R*	*R* ^2^	*F*	*β*	*t*	LLCI	ULCI
Young children’s digital addiction	Family socioeconomic status	0.160	0.026	10.603**	−0.160	−3.256**	−0.026	−0.006
Emotional warmth and understanding	Family socioeconomic status	0.155	0.024	9.814**	0.155	3.133**	0.351	1.533
Young children’s digital addiction	Family socioeconomic status	0.234	0.055	11.546***	−0.187	−3.801***	−0.028	−0.009
	Emotional warmth and understanding				0.172	3.492***	0.046	0.164
Over-intervention and over-protection	Family socioeconomic status	0.059	0.004	1.421	−0.059	−1.192	−0.888	0.218
Young children’s digital addiction	Family socioeconomic status	0.174	0.030	6.247**	−0.165	−3.335***	−0.026	−0.007
	Over-intervention and over-protection				−0.067	−1.367	−0.093	0.017
Rejection and denial	Family socioeconomic status	0.057	0.003	1.304	−0.057	−1.142	−0.850	0.225
Young children’s digital addiction	Family socioeconomic status	0.249	0.062	13.226***	−0.171	−3.533	−0.027	−0.008
	Rejection and denial				−0.191	−3.933	−0.157	−0.052
Punishment and harshness	Family socioeconomic status	0.157	0.025	10.166**	−0.157	−3.188**	−1.556	−0.369
Young children’s digital addiction	Family socioeconomic status	0.218	0.048	9.986***	−0.184	−3.724***	−0.028	−0.009
	Punishment and harshness				−0.150	−3.026**	−0.151	−0.032
Favoring subject	Family socioeconomic status	0.049	0.002	0.981	0.049	0.990	−0.308	0.932
Young children’s digital addiction	Family socioeconomic status	0.173	0.030	6.164**	−0.164	−3.320**	−0.026	−0.007
	Favoring subject				0.064	1.306	−0.021	0.102

^*^p < 0.05; **p < 0.01; ***p < 0.001.

A mediation model was used to test whether the effect of family socioeconomic status on young children’s digital addiction was produced through parenting styles. As shown in Table 3, mediation analysis showed that the mediating effect of parenting style on the relationship between family socioeconomic status and young children’s digital addiction was 0.006, 95% CI [0.003, 0.011]. The ratio of the indirect effect to the total impact was −37.5%. Thus, the association between family socioeconomic status and young children’s digital addiction can be partially explained by parenting styles.

**Table 3:** Total effect, direct effect, and total indirect effect.

Effect	Effect Value	SE	LLCI	ULCI
Total effect	−0.016	0.005	−0.026	−0.006
Direct effect	−0.022	0.005	−0.032	−0.013
Total Indirect effect	0.006	0.002	0.003	0.011

### 3.4 Testing for moderated mediation models

**Figure 2:**
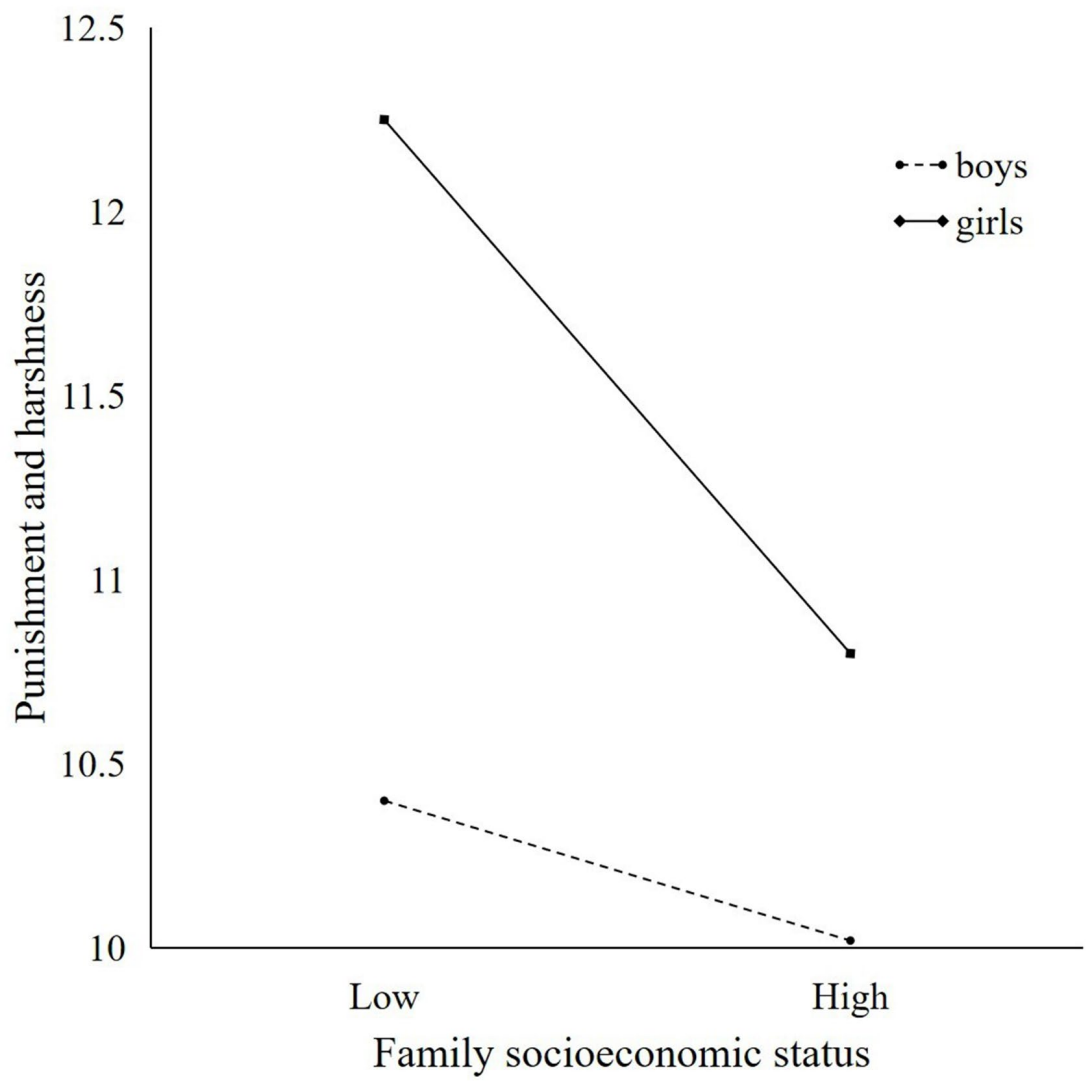
Relationship between punishment and harshness parenting style and family socioeconomic status.

The moderating role of young children’s gender in the first and second half paths of the model was analyzed through the PROCESS macro program. First, the moderating role of young children’s gender in the first half path was examined, and the results are shown in Table 4. Family socioeconomic status was a non-significant predictor of punishment and harshness parenting style (*β* = −0.042, *t* = −3.716, *p* > 0.05), young children’s gender was a significant predictor of punishment and harshness parenting style (*β* = −0.377, *t* = −2.316, *p* < 0.05), and the interaction term between family socioeconomic status and young children’s gender was a significant positive predictor of punishment and harshness parenting styles (*β* = 0.033, *t* = 2.040, *p* < 0.05), with 95% confidence intervals of [0.001, 0.064] and excluding 0, suggesting that young children’s gender moderates the relationship between family socioeconomic status and punishment and harshness parenting styles. Specifically, as shown in Figure 2, in the girls’ group, family socioeconomic status was a significant predictor of punishment and harshness parenting styles (*β* = −0.042, *p* < 0.001, 95% confidence interval [−0.064, −0.020]). In the boys’ group, family socioeconomic status was a non-significant predictor of punishment and harshness parenting styles (*β* = −0.009, *p* > 0.05, 95% confidence interval [−0.032, 0.013]).

**Table 4:** Moderating effect test for the first half of the path.

Variant	Punishment and harshness			
	*β*	SE	*t*	95%CI
Family socioeconomic status	−0.042***	0.011	−3.716	[−0.0641, -0.0198]
Young children’s gender	−0.377*	0.163	−2.316	[−0.697, -0.057]
Family socioeconomic status*Young children’s gender	0.033*	0.016	−2.04	[0.001, 0.064]
*R^2^*	0.039			
*F*	5.353**			

^*^p < 0.05; **p < 0.01; ***p < 0.001.

**Figure 3:**
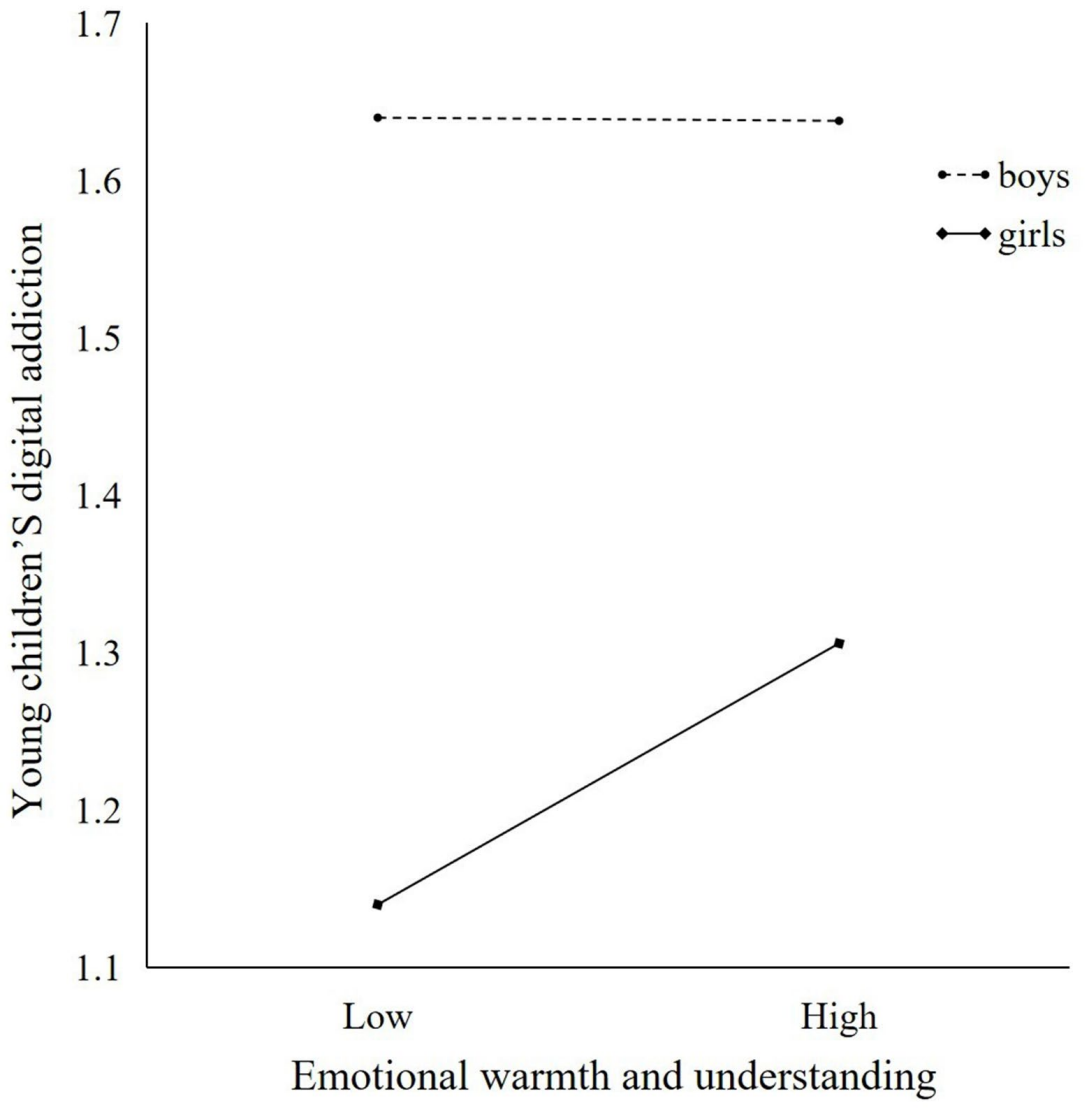
Relationship between young children’s digital addiction and emotional warmth and understanding parenting style.

Second, the moderating role of young children’s gender in the second half of the pathway was tested. As shown in Table 5. Emotional warmth and understanding parenting styles were significant predictors of digital addiction in young children (*β* = 0.165, *t* = 4.012, *p* < 0.001), gender of young children was an essential predictor of digital addiction (*β* = 0.497, *t* = −2.783, *p* < 0.01), and the interaction term between emotional warmth and understanding parenting styles and young children’s gender significantly and negatively predicted young children’s digital addiction (*β* = −0.166, *t* = −2.769, *p* < 0.01), with a 95% confidence interval of [−0.284, −0.048] and excluding 0, suggesting that young children’s gender moderates the relationship between emotional warmth and understanding parenting styles, and young children’s digital addiction. Specifically, as shown in Figure 3, in the group of girls, emotional warmth and understanding parenting styles were significant predictors of digital addiction (*β* = 0.165, *p* < 0.001, 95% confidence interval of [0.084, 0.246]). However, in the boys’ group, emotional warmth and understanding parenting style were not significant predictors of digital addiction (*β* = −0.001, *p* > 0.05, 95% confidence interval [−0.087, 0.085]).

**Table 5:** Moderating effects test for the second half of the path.

Variant	Young children’s digital addiction			
	*β*	*SE*	*t*	95%CI
Emotional warmth and understanding	0.165	0 0.041	4.012	[0.084, 0.246]
Young children’s gender	0.497**	0.179	−2.783	[0.146, 0.848]
Caring and understanding *Young children’s gender	−0.166**	0.06	−2.769	[−0.284, -0.048]
*R^2^*	0.039			
*F*	5.414**			

^*^p < 0.05; **p < 0.01; ***p < 0.001.

## 4 Discussion

This study found that family socioeconomic status significantly and negatively predicted digital addiction in young children, and the results validated research hypothesis 1. This conclusion is similar to the results of existing studies (Andreou and Svoli, 2013; Islam and Hossin, 2016; Malak et al., 2017). Ecosystem theory states that individuals live in interacting and interconnected environmental systems and that physiological and environmental factors can influence children’s psychological and behavioral development (Bronfenbrenner and Morris, 1998). The family’s socioeconomic status, as one of the essential components of the family environment system, is closely related to the development of good psychological quality and behavioral habits in young children. As mentioned in the theoretical model constructed by Urbanova et al. (2019), people with low socioeconomic status in the family tend to carry relatively more difficulties and less happiness. This state of life may expose them to more significant psychological stress and social barriers. As a result, individuals with low family socioeconomic status are more likely to seek comfort and escape from reality online. Some other researchers have pointed out that individuals with low socioeconomic status have a lower sense of self-worth (Twenge and Campbell, 2002), are more psychologically stressed (Kraus et al., 2011), have higher tendencies to anxiety and depression (Chen and Miller, 2013), higher impulsivity and lower inhibitory control (He and Yin, 2016), and are more prone to psychological problems and behavioral deviations than individuals in families with high socioeconomic status (Lynam et al., 2000; He and Yin, 2016). At the same time, previous studies have also found that individuals of low household socioeconomic status with lower inhibitory control, higher impulsivity, and higher stress are more inclined to overuse online social media (He et al., 2021). This may be because individuals with low inhibitory control have more difficulty controlling their impulses when confronted with online temptations. Thus, they are more likely to fall into a state of overuse. However, it is essential to emphasize that the relationship between self-regulation skills and digital addiction is not simply inverse. Moderate digital media use may also promote the development of self-regulation in individuals (Frost et al., 2019; Toh et al., 2023). For example, through online learning platforms or educational games, young children can learn how to control their behavior, make plans, and reach goals playfully, experiences that help them develop inhibitory control and other self-regulation skills. Thus, in further exploring the relationship between SES and digital addiction in the future, we cannot ignore the mediating role of self-regulatory capacity. Still, neither can we equate low inhibitory control with a high risk of digital addiction. Instead, we should focus on how education and interventions can enhance young children’s self-regulating ability in different family socioeconomic contexts, thereby reducing their risk of digital addiction.

The mediation analysis results indicated that parenting styles (emotional warmth and understanding, punishment and harshness) mediated the relationship between family socioeconomic status and young children’s digital addiction, and the results validated research hypothesis 2. This conclusion supports previous research findings. According to the ecosystem theory, family socioeconomic status belongs to the outer system, parenting style belongs to the microsystem, and family socioeconomic status needs to be passed through parenting style to act on the young children themselves (Bronfenbrenner, 1986). Some studies have found that parents of lower family socioeconomic status are more likely to use harsh, more authoritarian parenting styles, such as punishment and harshness and lack of communication (Hoffman, 2003). This parenting style may make young children more likely to exhibit poor self-control and disobedient behavior, leading to digital addiction (Zhang, 2016). It has also been found that parents in low-income families experience more stress and fatigue, are less responsive to their young children’s needs, and allocate less time management and energy to their young children’s use of media, resulting in higher rates of digital addiction among their children, compared to higher-income families (Livingstone et al., 2015). This research conclusion supports the family investment theory, in which parents whose families have a higher socioeconomic status are more likely to invest in early childhood education, which includes resources such as time, participation in activities, and parenting styles, in addition to financial resources (Schofield et al., 2011; Layte, 2017). Specifically, parents whose families have higher socioeconomic status are more willing to participate in their children’s daily life and academic activities and are more inclined to use discussion to solve problems with their children. Conversely, parents from lower socioeconomic status families have to work longer hours for economic reasons and spend less time with their young children, leading to increased problematic behaviors (Gennetian and Rodrigues, 2021). This finding also supports the family stress theory that parents with lower socioeconomic status in the family are more prone to financial stress and psychological problems due to lack of insufficient financial income, which makes them tend to display harsh and punitive parenting styles that negatively affect the physical and mental development of young children and increase their tendency to become addicted to the Internet (Conger and Conger, 2002). In addition, parents of low family socioeconomic status may be unduly influenced by adverse media reports on the impact of Internet use on children’s behavior. Due to their low level of education and lack of experience with the Internet, they may strictly control or prohibit Internet use (Álvarez et al., 2013). This approach may foster children’s aversion and rejection of their parents, exacerbate excessive Internet use, and generate digital addiction (Li et al., 2016). This will create a vicious circle. However, in families with high family socioeconomic status, most parents are well-educated, have extensive online skills, understand the positive and negative impacts of the Internet, and consciously teach their children how to use the Internet (Álvarez et al., 2013), which can increase the breadth and depth of parent–child communication and improve parent–child relationships. At the same time, it can avoid some of the risks of Internet use and reduce the probability of digital addiction (Lee and Chae, 2007; Dong et al., 2021).

The results of the moderation analysis indicated that family socioeconomic status and punishment and harshness parenting styles were moderated by gender, and the results supported hypothesis 3. This conclusion is similar to the results of previous studies. The different expectations placed on different genders under traditional attitudes result in a “Son preference” mentality (Wu et al., 2013) that parents are more inclined to satisfy boys at the expense of girls when resources are scarce (Hannum et al., 2009). Currently, this phenomenon still exists among families of lower socioeconomic status (Yang et al., 2016), with parents striving to satisfy boys’ interests and over-protection and spoiled upbringing with adequate resources for the child. In contrast, girls are more likely to be taught in a critical, punishment, and severity manner (Zhang and Ma, 2019). The present study found no significant difference in digital addiction in young children by gender. Although studies have shown that boys have significantly higher levels of digital addiction than girls, some studies point to a narrowing of this gap (Weiser, 2000). It is worth exploring that the present study found a significant gender difference in the relationship between caring and understanding parenting style and young children’s digital addiction, and girls’ parents’ caring and understanding were more predictive of young children’s digital addiction compared to boys’. This result supports hypothesis 4. However, the conclusions of this study differ from the views of previous scholars, namely Niu et al. (2023) noted that in the adolescent population, the use of positive parenting styles by parents of boys was a more significant negative predictor of digital addiction compared to girls. This difference may be due to differences in the characteristics of the subject sample, with young children aged 3–6 years being more curious but less self-controlled compared to adolescents. Emotional warmth and understanding parents may be overly tolerant and understanding and lack supervision and control over their children’s activities and behaviors (Anandari, 2016), which may lead to overindulgence in the online world of digital addiction and lead to digital addiction. In addition, scholars have noted that girls are more susceptible to parenting styles to parenting styles because girls feel more lonely than boys and are more likely to use the Internet in search of socialization and recognition, which leads to a rise in their risk of digital addiction (Akhter et al., 2020; Guo et al., 2023).

## 5 Implications and limitations

### 5.1 Theoretical and practical implications

This study explores the influencing factors and internal mechanisms of digital addiction for the first time for a group of young children, filling the gap in the field of digital addiction research for this age group and expanding the scope of the target group of digital addiction research. Second, the study clarified the negative correlation between family socioeconomic status and young children’s digital addiction and explored the moderating role of parenting styles as well as the moderating role of gender. These findings emphasize the critical role of the family environment in young children’s development and provide a theoretical rationale for early intervention, as well as directing policy and legislative attention to young children’s digital health. Third, the study reveals the influence of factors such as family socioeconomic status, parenting style, and the gender of young children on young children’s digital addiction, which provides new perspectives and a theoretical basis for understanding the formation mechanism of young children’s digital addiction. In addition, the research results not only help to improve the existing theoretical model of young children’s digital addiction but also provide a reference for future research on young children’s digital addiction and related fields.

The study found that family socioeconomic status and parenting styles are influential factors in young children’s digital addiction and that young children’s gender plays an essential moderating role in this influential mechanism. Given this, the study recommends the following practices to prevent and intervene in digital addiction among young children. First, the Government and social organizations should strengthen special funds and educational subsidies to enhance family socioeconomic support and educational resources. AI technology can accurately identify the needs of families with low socioeconomic status and provide personalized economic assistance and education programs. At the same time, community support networks should be established to promote sharing of parental experience and optimize the environment for family growth. Second, the education and health sectors should jointly organize lectures and workshops to popularize caring and understanding parenting styles, reduce punitive education, and enhance parents’ communication and emotion management abilities. AI home education assistants can be introduced to provide customized parenting advice and enhance parents’ understanding of their children’s emotions through emotion recognition technology. Encourage non-screen time parent–child activities, such as outdoor adventure and parent–child reading, to improve parent–child relationships. Finally, analyze the different manifestations of gender in young children’s use of numbers and design gender-appropriate non-numeric activities to reduce number dependence. Strengthen gender-sensitive education for parents and guide them to respond to their children’s specific digital behaviors effectively. In addition, personalized analysis using AI technology can accurately identify and intervene in young children’s risk of digital addiction, ensuring that interventions are more targeted and effective.

### 5.2 Limitations and future research

This study has many contributions and implications but is not without shortcomings. First, the study used a cross-sectional research design, which could not show the dynamic relationship between family socioeconomic status, parenting styles, and young children’s digital addiction. In the future, we will observe the changes in family socioeconomic status, parenting styles, and young children’s digital addiction behaviors at different points in time through a long-term follow-up survey to reveal the dynamic relationship between them. Second, the variables of young children’s digital addiction and parenting styles in the study were reported by parents. Although it has been found through testing that there is no serious issue of common method bias in the data of this study, multiple ways of collecting data should be considered in future research to enhance the objectivity of conclusions. Second, this study only examined the relationship between family socioeconomic status, parenting styles, and young children’s digital addiction. However, other studies have shown that factors such as self-regulation, inhibitory control, parental digital addiction, parental mental health status, and parent–child relationships also affect young children’s digital addiction (Frost et al., 2019; Lam, 2020; Li et al., 2022; Toh et al., 2023). In addition, it has been noted that digital addiction can, in turn, affect parenting styles (Dong et al., 2021). Therefore, in future studies, we can consider adding variables such as parental digital addiction, parental mental health status, and parent–child relationship and exploring bidirectional influence pathways to more accurately reflect the intrinsic mechanisms of the factors affecting young children’s digital addiction.

## 6 Conclusion

This study explored the factors influencing digital addiction in young children and the internal mechanisms between them and found that family socioeconomic status was significantly negatively correlated with digital addiction in young children. Parenting styles (caring and understanding and punishment and severity) mediate the relationship between family socioeconomic status and young children’s digital addiction. Early childhood gender moderates the relationship between family socioeconomic status, punishment, severity parenting styles, caring and understanding parenting styles, and early childhood digital addiction. This finding contributes to further understanding and knowledge of the relationship between family socioeconomic status, parenting styles, gender of young children, and young children’s digital addictions, which is of great significance to the prevention of young children’s digital addiction and the promotion of young children’s healthy physical and mental development.

## Data Availability

The data analyzed in this study is subject to the following licenses/restrictions: The data presented in this study are available on request from the corresponding author. The data are not publicly available due to ethical requirements. Requests to access these datasets should be directed to HL, huanhuanli@xjnu.edu.cn.
